# Non-stop mRNA decay: a special attribute of *trans-*translation mediated ribosome rescue

**DOI:** 10.3389/fmicb.2014.00093

**Published:** 2014-03-11

**Authors:** Krithika Venkataraman, Kip E. Guja, Miguel Garcia-Diaz, A. Wali Karzai

**Affiliations:** ^1^Department of Biochemistry and Cell Biology, Stony Brook UniversityStony Brook, NY, USA; ^2^Center for Infectious Diseases, Stony Brook UniversityStony Brook, NY, USA; ^3^Department of Pharmacological Sciences, Stony Brook UniversityStony Brook, NY, USA

**Keywords:** RNase R, tmRNA, SmpB, non-stop mRNA, *trans-*translation

## Abstract

Decoding of aberrant mRNAs leads to unproductive ribosome stalling and sequestration of components of the translation machinery. Bacteria have evolved three seemingly independent pathways to resolve stalled translation complexes. The *trans-*translation process, orchestrated by the hybrid transfer-messenger RNA (tmRNA) and its essential protein co-factor, small protein B (SmpB), is the principal translation quality control system for rescuing unproductively stalled ribosomes. Two specialized alternative rescue pathways, coordinated by ArfA and ArfB, have been recently discovered. The SmpB-tmRNA mediated *trans-*translation pathway, in addition to re-mobilizing stalled translation complexes, co-translationally appends a degradation tag to the associated nascent polypeptides, marking them for proteolysis by various cellular proteases. Another unique feature of *trans*-translation, not shared by the alternative rescue pathways, is the facility to recruit ribonuclease R (RNase R) for targeted degradation of non-stop mRNAs, thus preventing further futile cycles of translation. The distinct C-terminal lysine-rich (K-rich) domain of RNase R is essential for its recruitment to stalled ribosomes. To gain new insights into the structure and function of RNase R, we investigated its global architecture, the spatial arrangement of its distinct domains, and the identities of key functional residues in its unique K-rich domain. Small-angle X-ray scattering models of RNase R reveal a tri-lobed structure with flexible N- and C-terminal domains, and suggest intimate contacts between the K-rich domain and the catalytic core of the enzyme. Alanine-scanning mutagenesis of the K-rich domain, in the region spanning residues 735 and 750, has uncovered the precise amino acid determinants required for the productive engagement of RNase R on tmRNA-rescued ribosomes. Theses analyses demonstrate that alanine substitution of conserved residues E740 and K741**result in profound defects, not only in the recruitment of RNase R to rescued ribosomes but also in the targeted decay of non-stop mRNAs. Additionally, an RNase R variant with alanine substitution at residues K749 and K750 exhibits extensive defects in ribosome enrichment and non-stop mRNA decay. In contrast, alanine substitution of additional conserved residues in this region has no effect on the known functions of RNase R. *In vitro* RNA degradation assays demonstrate that the consequential substitutions (RNase R^E740A/K741A^ and RNase R^K749A/K750A^) do not affect the ability of the enzyme to degrade structured RNAs, indicating that the observed defect is specific to the *trans-*translation related activities of RNase R. Taken together, these findings shed new light on the global architecture of RNase R and provide new details of how this versatile RNase effectuates non-stop mRNA decay on tmRNA-rescued ribosomes.

## INTRODUCTION

Controlling mRNA stability is one of the key means of post-transcriptional regulation of gene expression. A major factor contributing to transcript stability is ribosome-mediated protection of potential cleavage sites from the endonucleolytic activity of RNase E (Carpousis, 2007), an essential component of the RNA degradosome. Translating ribosomes are thought to sterically hinder ribonucleases from accessing cleavage sites present in mRNAs, leading to their increased stability (Carpousis, 2007; Bouvier and Carpousis, 2011). However, the stability of defective or non-stop mRNAs, which promote the accumulation of unproductively stalled ribosomes, is dramatically reduced. In bacteria, a universally conserved ribosome rescue mechanism called *trans*-translation facilitates the selective increase in non-stop mRNA decay (Karzai et al., 2000; Withey and Friedman, 2003; Dulebohn et al., 2007; Keiler, 2008; Wower et al., 2008; Hayes and Keiler, 2010; Barends et al., 2011). The central components orchestrating the *trans*-translation process are the hybrid transfer-messenger RNA (tmRNA; Keiler et al., 1996) and its requisite protein cofactor SmpB (Karzai et al., 1999). The biological significance of salvaging unproductively stalled ribosomes is highlighted by the fact that three independent ribosome rescue mechanisms have evolved in *E*.* coli *and many related bacterial species. However, selective non-stop mRNA decay is a unique consequence of the SmpB-tmRNA mediated *trans*-translation process (Richards et al., 2006; Dulebohn et al., 2007; Ge et al., 2010; Liang and Deutscher, 2012; Camenares et al., 2013) – an equivalent mRNA decay process has not been identified for the alternate ArfA and ArfB ribosome rescue pathways (Chadani et al., 2010, 2011, 2012).

The highly conserved ribonuclease R (RNase R) is the sole 3′–5′ exoribonuclease responsible for the selective degradation of non-stop and defective mRNAs (Richards et al., 2006). RNase R belongs to the RNB family of exoribonucleases and shares ~45% sequence similarity with RNase II. Based on sequence analysis, it was inferred that RNase R and RNase II have similar overall organization of the cold shock, catalytic RNB, and S1 domains (Zuo and Deutscher, 2001). In addition to these core domains, RNase R has distinctive N-terminal helix-turn-helix and C-terminal lysine-rich (K-rich) domains**that are not present in RNase II. Recent data have made it abundantly clear that the activities of RNase R are intimately linked with the SmpB-tmRNA system. While RNase R is known to participate in the *trans*-translation process (Karzai and Sauer, 2001; Richards et al., 2006) and play a decisive role in the cell-cycle dependent degradation of circularly permuted tmRNA (Hong et al., 2005), the SmpB-tmRNA complex also somehow regulates the stability of RNase R (Liang and Deutscher, 2012). Despite the high degree of similarities between RNase II and RNase R, the SmpB-tmRNA system does not appear to affect the function or stability of RNase II, suggesting the unique N- and C-terminal domains of RNase R are involved in the specialized functions of this versatile enzyme. Indeed, we recently demonstrated that the K-rich C-terminal domain of RNase R is required for the tmRNA-mediated ribosome enrichment and non-stop mRNA decay activities of RNase R (Ge et al., 2010).

While the crystal structure of RNase II has revealed its structural organization at atomic resolution (Frazao et al., 2006; Zuo et al., 2006), information on the three-dimensional (3D) shape and domain architecture of RNase R is not available. Thus, it is of great interest to gain new insights into the overall 3D shape and spatial domain organization of RNase R and identify key functional residues that are required for its *trans*-translation related activities. In this study, we used small-angle X-ray scattering (SAXS) techniques to determine the global solution structure of RNase R. Our analysis reveals the overall architecture of RNase R, the high degree of similarity of its core domain to RNase II, and the relative spatial positioning of the flexible N- and C-terminal domains with respect to its catalytic core. Additionally, systematic mutational and biochemical analyses of the K-rich domain of RNase R highlight the importance of residues E740–K741 and K749–K750 in engaging tmRNA-rescued ribosomes and facilitating selective degradation of non-stop mRNAs.

## MATERIALS AND METHODS

### STRAINS AND PLASMIDS

*Escherichica coli* strain MG1655 *rnr::kan* was obtained by P1 transduction using the Keio *rnr::kan *disruption strain (Baba et al., 2006) as a donor. A plasmid expressing the coding region of N-terminal domain of λ-cI protein (pλ-*cI*-NS), which encodes for an N-terminal His6 epitope but lacks in-frame stop codons, served as the source of the non-stop reporter mRNA. A control reporter plasmid (pλ-*cI*-S), which encodes for an N-terminal His6 epitope and has two tandem in-frame stop codons, served as the source of the “normal” or stop codon containing reporter mRNA. A pACYCDuet-1 plasmid (p*rnr*) harboring the *E*.* coli* *rnr *gene under the control of the arabinose inducible P_BAD_ promoter was used as a template to synthesize RNase R variants by site directed mutagenesis. For purification of RNase R and its variants, the coding region of *E*.* coli* *rnr *gene**was cloned into the MCS of pET15b under the control of isopropyl β-D-thiogalactoside (IPTG)-inducible T7 promoter. This plasmid was used as a template to synthesize RNase R variants by site directed mutagenesis. The plasmids were transformed into W3110 (DE3) *rnr* *rnb* strain for over-expression and purification of the corresponding RNase R variants, as previously described (Ge et al., 2010).

### SAXS MEASUREMENTS

Purified RNase R samples were prepared in 50 mM Tris-HCl (pH 7.5), 250 mM KCl, and 1 mM DTT within 24 h of data acquisition and stored at 4^°^C. Scattering data were collected at beamline X9 of the National Synchrotron Light Source (NSLS, Upton, NY, USA) using a Pilatus 300K located 3.4 m from the sample for small-angle X-ray data. Wide-angle X-ray scattering data were collected simultaneously with SAXS data using a Photonic Science CCD camera located 0.47 m from the sample. Twenty microliters of sample were continuously flowed through a 1 mm diameter capillary and exposed to a 400 μm × 200 μm X-ray beam with a wavelength of 0.9184 Å for 30 s of total measurement time. Scattering data were collected at concentrations of 2.2, 3.7, 4.2, 5.0, and 10 mg/mL for RNase R^WT^ and at concentrations of 0.7, 2.8, 3.0, 3.5, and 6.0 mg/mL for RNase R^723^. Each concentration was measured in triplicate. Normalization for beam intensity, buffer subtraction, and merging of data from both detectors were carried out using PRIMUS (Konarev et al., 2003). The radius of gyration (*R*_g_) was calculated using a Guinier approximation, I(q)=I(0)exp(-q^2^ R_g_^2^ /3), where a plot of *I*(*q*) and *q*^2^ is linear for *q* <1.3/*R*_g_ (Guinier, 1939). Three independent scattering trials were averaged. GNOM was used to determine the pair distribution function, *P*(*r*), and maximum particle dimension, *D*_max_ (Semenyuk and Svergun, 1991). The linearity of the Guinier region and the forward scattering intensity were used to validate that the samples were monodisperse in solution. The forward scattering intensity, *I(0)*, is the theoretical scattering at a *q* value of 0 and is proportional to the molecular weight of the sample (Putnam et al., 2007). *I(0)*/*c*, where *c* is sample concentration, was identical for all RNase R measurements. For RNase R^723^, ten independent *ab initio* beads models that describe the experimental data were calculated using DAMMIN (Svergun, 1999). The resulting models, which had an average χ^2^ of 0.65 ± 0.16, and a normalized spatial discrepancy (NSD) of 0.71 Å, were subsequently aligned, averaged, and filtered by occupancy using the DAMAVER suite of programs (Volkov and Svergun, 2003). An electron density map was calculated from the filtered average model using SASTBX (Liu et al., 2012). For RNase R^WT^, the crystal structure of RNase II was modeled against the solution structure data by combined rigid body fitting and *ab initio* bead modeling, using BUNCH (Petoukhov and Svergun, 2005).

### REPORTER mRNA STABILITY AND RIBOSOME ENRICHMENT ANALYSIS

*Escherichica coli* strain MG1655 *rnr::kan *harboring the reporter plasmid pλ-*cI*-NS and the various p*rnr* variants were grown at 37^°^C in Luria-Bertani (LB) broth containing 0.01% arabinose, 100 μg/ml of Ampicillin, and 30 μg/ml of Chloramphenicol. At an optical density at 600 nm (OD_600_) of ~0.6, expression of the λ-*cI*-NS reporter mRNA was induced with a final concentration of 1 mM IPTG for 1 h. Equal number of cells were harvested and total RNA was isolated using TRI Reagent (MRC Inc.). To test the activity of RNase R variants, *in vitro *RNA degradation assays were performed as previously described (Ge et al., 2010). The oligoribonucleotide substrate used was a single stranded RNA with an internal step-loop structure and a 3′ poly A overhang (*trp*At-A_10_: 5′-GCAGCCCGCCUAAUGAGCGGGCAAAAAAAAAA-3′). Reaction mixtures containing the 32P-labeled *trp*At-A_10_ substrate and the desired RNase R variants were assembled and incubated at 37^°^C. Aliquots of 5 μl were taken at indicated time points and quenched by the addition of 5 μl of the formaldehyde gel-loading dye. Full-descriptions of the *in vivo* reporter mRNA stability and ribosome enrichment assays have been described elsewhere (Ge et al., 2010; Mehta et al., 2012). A brief description of the ribosome enrichment assay is provided here. Non-stop or stop reporter mRNAs were expressed in MG1655 *rnr::kan *strain complemented with designated plasmid borne RNase R variant, under a P_BAD_ promoter. Cells were grown in media containing 0.01% arabinose to OD_600_ of 1.0 and the expression of reporter constructs was induced by the addition of IPTG to a final concentration of 1 mM. Cells were allowed to grow for another 45 min, harvested, resuspended in Buffer E (50 mM Tris-HCl pH 7.5, 300 mM NH_4_Cl, 20 mM MgCl_2_, 2 mM β-ME, and 10 mM Imidazole) and lysed by French Press. Ribosomes were pelleted from cleared cell lysates by centrifugation at 29,000 rpm for 16–18 h through a 32% sucrose cushion in Buffer E. Ribosomes translating the reporter mRNAs were purified from tight-coupled ribosomes by Ni^2^^+^-NTA affinity chromatography. The ribosome-associated RNase R was detected by Western blot analysis using polyclonal anti-RNase R antibody. Protein bands were quantified using ImageJ.

## RESULTS

### GLOBAL ARCHITECTURE AND DOMAIN ORIENTATION OF RNase R IN THREE-DIMENSIONAL SPACE

**FIGURE 1:**
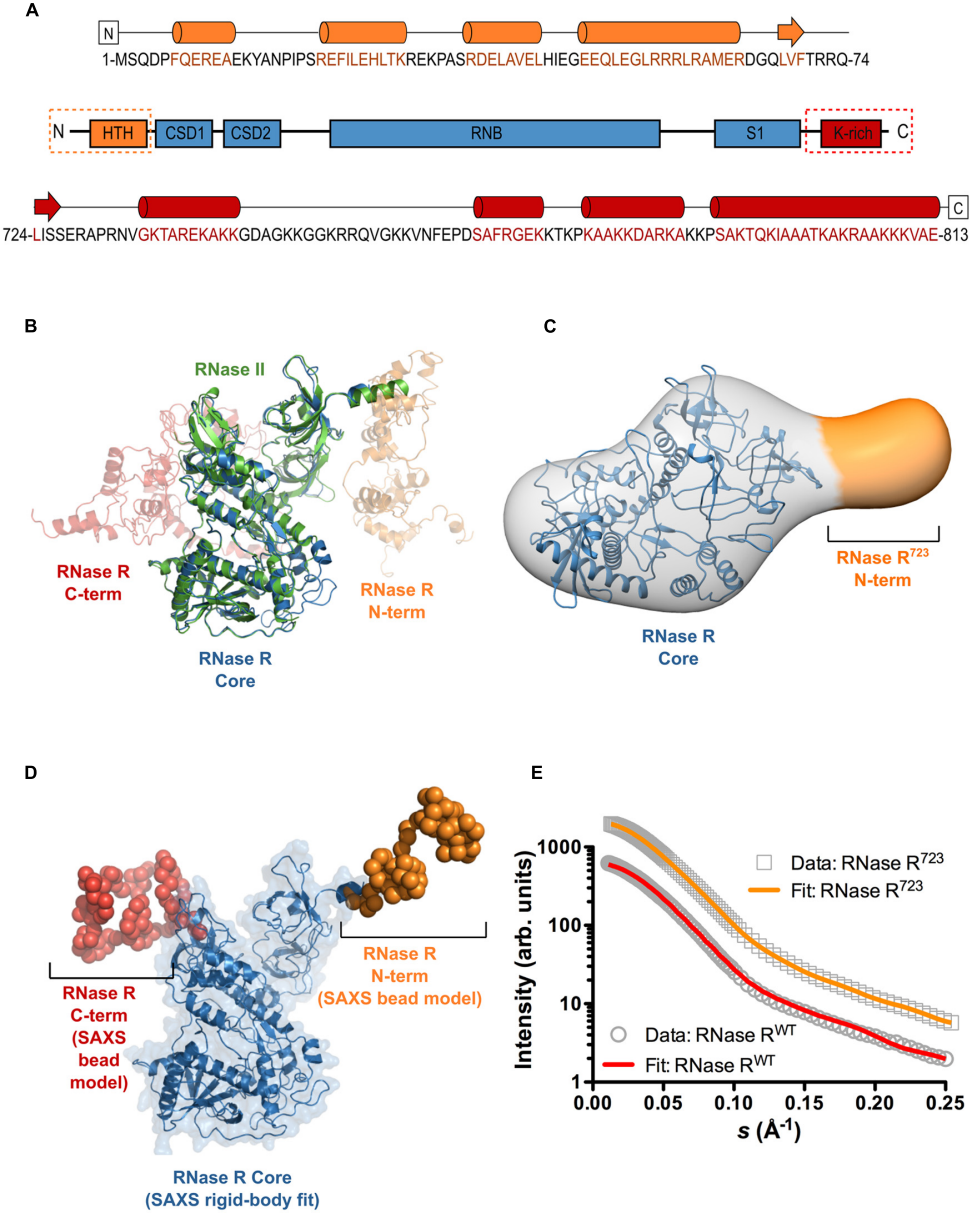
**The overall shape and relative domain orientation of full-length RNase R.** **(A)** Secondary structure prediction from I-TASSER for the N-terminal domain (orange), the catalytic core (blue), and the C-terminal domain (red) of RNase R. Predicated helical regions are shown as cylinders, β-strands as arrows, and random coil regions as black lines. **(B)** Super-position of the crystal structure of RNase II (PDB ID 2ID0) and the three top-scoring homology models of full-length RNase R generated with I-TASSER. RNase II is shown as a green cartoon, the conserved core of RNase R is shown as a blue cartoon. **(C)** SAXS envelope showing the 3D molecular shape of RNase R^723^. The I-TASSER model of the conserved core domain (shown in panel **B**) has been docked into the SAXS envelope using SITUS, with a resulting correlation coefficient of 0.88. The additional density seen in the SAXS envelope (indicated in orange) corresponds to residues 1–65 of RNase R^723^. The C-terminal residue in the I-TASSER model of the core domain is indicated with an asterisk. **(D)** SAXS model of RNase R^WT^ shows the 3D shape and relative orientations of the C- and N-terminal domains. The core domain was fit to the SAXS data as a rigid body (shown as a blue cartoon with transparent surface), and the missing terminal regions were modeled as C-α beads using the BUNCH program of the ATSAS suite. **(E)** The experimental scattering profiles of RNase R^WT^ and RNase R^723^. The relative scattering intensity (*I*) is plotted as a function of the momentum transfer, or scattering vector, *s* (*s* = 4πsinθ/λ where λ is the beam wavelength and θ is the scattering angle). The raw data for RNase R^723^ and RNase R^WT^ are shown as gray circles and rectangles, respectively. Simulated scattering curves that correspond to the SAXS envelope for the RNase R^723^ (shown in panel **C**) and the BUNCH model for RNase R^WT^ (shown in panel **D**) are shown as solid orange and red lines, respectively. The discrepancy between the raw and simulated scattering data is indicated by the value of χ^2^. The small χ^2^ value observed (1.43 for RNase R^WT^ and 0.68 for RNase R^723^) demonstrates a robust fit.

To gain insight into the overall 3D architecture and relative domain positioning of RNase R, we determined its solution structure at low resolution using SAXS techniques. As a preliminary step, and as a complement to the low resolution SAXS data, we used the I-TASSER platform (Zhang, 2008), employing the entire protein data bank (PDB) as the search space, to generate a homology model of full-length RNase R (RNase R^WT^). Our results provide secondary structure predictions for the N- and C-terminal domains of RNase R (**Figure 1A**) that are in good agreement with previous studies (Matos et al., 2011). The three top scoring models from I-TASSER (**Figure 1B**) support the notion that RNase R has a structurally conserved core domain that is highly similar to the known crystal structure of RNase II (Frazao et al., 2006; Zuo et al., 2006). The I-TASSER models predicted that the unique N- and C-terminal domains, which do not share significant homology to any known structures, flank the core catalytic domain of RNase R. Notably, the C-terminal K-rich domain is predicted to contain an extensive loop segment that likely imparts significant flexibility to this domain. To further characterize the 3D shape and spatial arrangement of the N- and C-terminal domains, we collected SAXS data on RNase R^WT^ and RNase R^723^, an RNase R variant lacking the entire K-rich domain. The SAXS envelope for RNase R^723^ has a shape that is highly similar to RNase II. Docking of the conserved core domain into the SAXS envelope using SITUS resulted in a robust fit with a correlation coefficient of 0.88 (**Figure 1C**). Additional density is apparent near the N-terminus that most likely corresponds to residues 1–65 of RNase R. Having confirmed that the solution structure of RNase R displays a conserved molecular shape with RNase II, we collected SAXS data on RNase R^WT^, and carried out rigid-body modeling with the conserved core domain of RNase R. We combined the core domain modeling with *ab initio* C-α bead modeling for the missing N- and C-terminal domains using BUNCH (**Figure 1D**). The resulting SAXS model of RNase R^WT^ reveals a tri-lobed structure that is somewhat elongated, and is consistent with the presence of extensive flexible loops, as predicted by homology modeling. The scattering patterns from RNase R^723^ and RNase R^WT^ (**Figure 1E**) were quite similar, reflecting their largely analogous topologies, and the SAXS model of the N-terminal and core domains of RNase R^WT^ correlated well with the overall shape of the RNase R^723^ envelope. Both models demonstrate an excellent fit to the raw scattering data (**Figure 1E**), with discrepancies (χ^2^) of 0.68 for RNase R^723^ and 1.43 for RNase R^WT^. Visual comparison of the two models reveals the relative positioning of the K-rich C-terminal lobe with respect to the core and N-terminal domains. Both the I-TASSER and SAXS models imply intimately close links between the catalytic core and the K-rich domains of RNase R, suggesting a potential regulatory role for the K-rich domain. However, the extent of such contacts and the identity of functionally important amino acid residues in the K-rich domain that participate in any potential intra- or inter-molecular interactions have not been determined.

### IDENTIFICATION OF FUNCTIONALLY IMPORTANT RESIDUES IN THE K-rich DOMAIN OF RNase R

**FIGURE 2:**
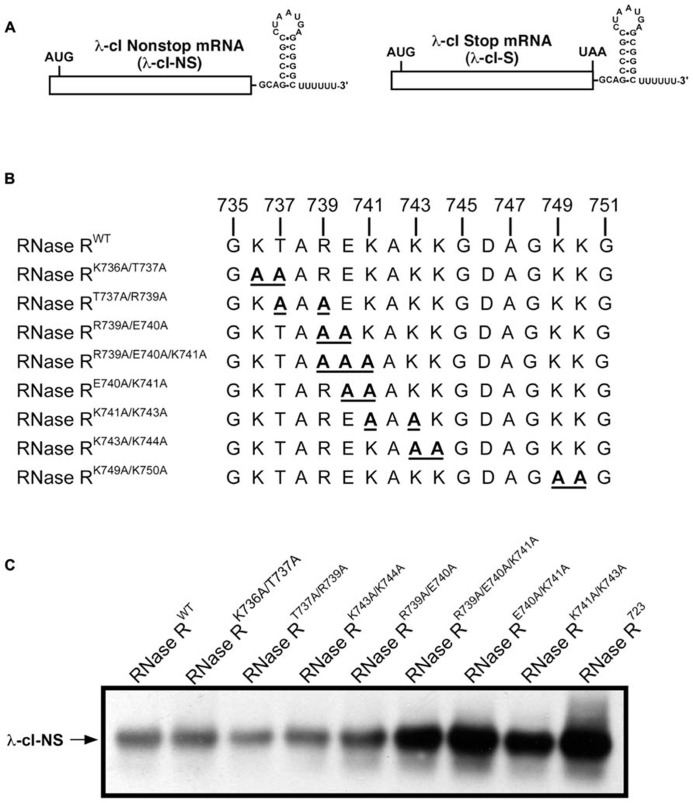
**Steady state level of non-stop reporter mRNA.** **(A)** A schematic representation of the reporter mRNAs used in this study. The λ-cI non-stop reporter (λ-cI-NS) is composed of the coding region of λ-cI N-terminal domain (depicted as a rectangle) followed by the transcriptional terminator (*trp*At nucleotide sequence at the 3′ end). The corresponding λ-cI stop reporter (λ-cI-S) has an in-frame UAA stop codon before the transcriptional terminator. (**B)** A representation of alanine substitutions in the region between amino acids 735 and 750 of the K-rich domain. (**C)** A representative Northern blot showing the effect of alanine substitutions in the region between residues 735 and 750 on non-stop mRNA accumulation. Cells co-expressing the λ-cI-NS reporter with indicated plasmid-borne RNase R variants were harvested. Total RNA samples isolated from equal number of cells were resolved by electrophoresis on denaturing formaldehyde agarose gels followed by Northern blot analysis with a probe specific for the reporter RNA. Alanine substitutions at residues E740 and K741 exhibit significant defect in non-stop mRNA degradation.

In an effort to uncover the identities of functionally important residues in the K-rich domain, we generated a series of alanine substitution variants of RNase R. We were guided by previous studies of the K-rich domain, which demonstrated that C-terminal truncations from residue 813 to 750 (RNase R^750^) had no effect on the *trans-*translation related activity of RNase R (Ge et al., 2010). These studies also showed that a further truncation of 15 residues in the K-rich domain, to amino acid residue 735 (RNase R^735^), resulted in a discernible defect in the ability of the enzyme to degrade non-stop mRNA. RNase R truncation variant RNase R^723^, which lacks the entire K-rich region, exhibited a similar pronounced defect in degradation of non-stop mRNA (Ge et al., 2010). Based on these results, we reasoned that some of the critical amino acid residues, responsible for the *trans-*translation activity of RNase R, must lie in the region encompassing residues 735 to 750. To evaluate the validity of this inference, we performed alanine-scanning mutagenesis of conserved amino acid residues in this region and assessed their contribution to the SmpB-tmRNA mediated targeted degradation of non-stop mRNA. To facilitate this assessment, we used plasmid-encoded reporter transcripts that are comprised of the coding sequence for the N-terminal domain of the bacteriophage λ cI gene followed by the *trp* operon transcriptional terminator (*trp*At). To ensure evaluation of a non-stop mRNA specific process, we utilized two related versions of this reporter (**Figure 2A**), a non-stop transcript lacking in-frame stop codons (λ-cI-NS), and a “normal” control transcript possessing an in-frame stop codon (λ-cI-S). The λ-cI-NS non-stop transcript has been well characterized and is known to promote ribosome stalling at its 3′ end, whereas the stop codon containing λ-cI-S variant is readily translated and promotes efficient recycling of the translation machinery (Sundermeier et al., 2008; Mehta et al., 2012). The competence of RNase R alanine substitution variants in reporter mRNA decay was analyzed in a strain lacking chromosomal *rnr* and bearing plasmids for controlled expression of both the reporter transcript and one of the RNase R alanine substitution variants. Expression of RNase R, and its variants, was from a pACYCDuet-based plasmid bearing the *rnr* coding sequence under the control of the arabinose inducible P_BAD_ promoter.

**FIGURE 3:**
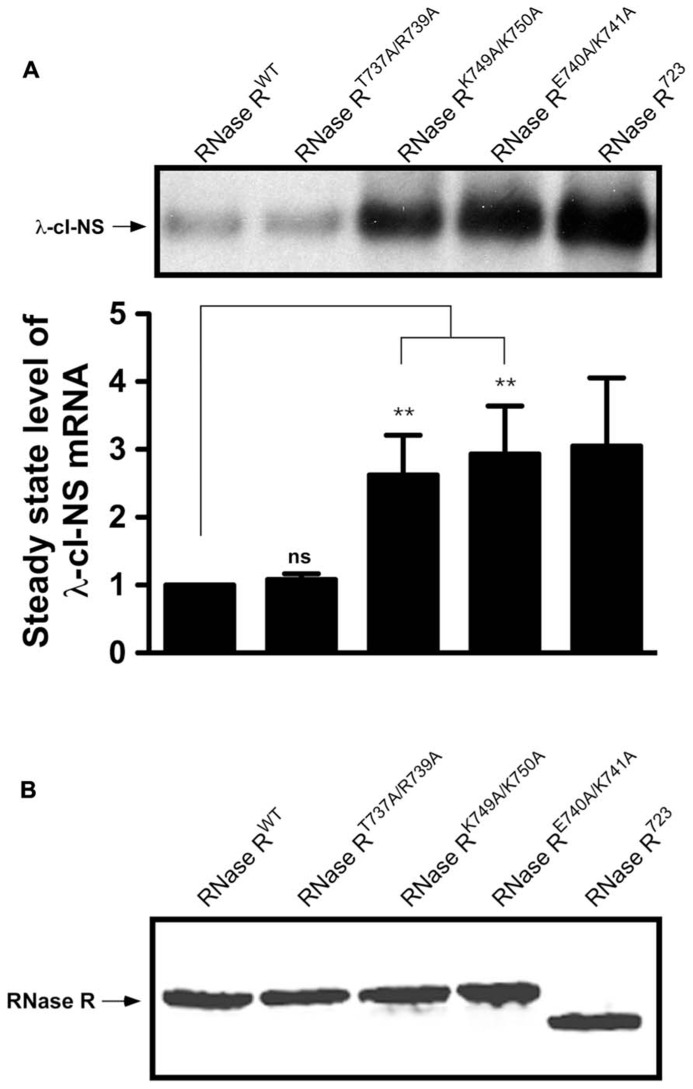
**Non-stop reporter mRNA accumulates in the presence of RNase R^**K749A/K750A**^, and RNase R^**E740A/K741A**^.** **(A)** A representative Northern blot showing the steady state level of non-stop mRNA in the presence of RNase R^T737A/R739A^, RNase R^K749A/K750A^, and RNase R^E740A/K741A^. The latter two variants are defective in non-stop mRNA degradation. *P*-values were calculated by performing student’s *t*-test analysis with RNase R^WT^ and RNase R^K749A/K750A^ (^**^P <0.005) or RNase R^WT^ and RNase R^E740A/K741A^ (^**^P <0.003). The graph represents the fold change in the steady state level of λ-cI-NS mRNA in the presence of indicated RNase R variants with respect to RNase R^WT^. The data are representative of three independent experiments (MEAN ± SEM, standard error of the mean). ns = not statistically significant. **(B)** Steady state levels of RNase R variants are similar. A representative Western blot examining the relative steady state levels of RNase R and its alanine-substitution variants is shown. MG1655 *rnr *strain harboring each p*rnr *variant**plasmid was grown in LB with 0.01% arabinose and 30 μg/ml of chloramphenicol. Equal numbers of cells were harvested in mid-log phase and processed for Western blot analysis, using RNase R specific antibodies.

We expressed each RNase R alanine substitution variant alongside the λ-cI-NS reporter mRNA, and measured the relative steady state abundance of the non-stop transcript. For all mRNA stability assessments, we used RNase R^WT^ as a positive control and the previously validated RNase R^723^ truncation variant (Ge et al., 2010) as a negative control. This analysis showed that various combinations of single and double alanine substitutions at residues K736, T737, and R739 had little or no effect on the steady state level of the reporter mRNA (**Figures 2B,C**). Similarly, single and double alanine substitutions at residues K743 and K744 had no effect on the steady state level of the non-stop reporter mRNA (**Figure 2C**). Alanine substitution at E740 or K741, either individually or in combination with other neighboring residues (RNase R^R739A/E740A^ and RNase R^K741A/K742A^), resulted in measurable increases in the accumulation of non-stop mRNA (**Figure 2C**), indicating a functional role for these adjoining residues in the mRNA decay activity of RNase R. To further investigate their functional contributions, we generated RNase R^E740A/K741A^, a double alanine substitution variant at residues E740 and K741. Assessment of RNase R^E740A/K741A^ revealed profound defects in non-stop mRNA degradation (**Figure 2C**). Alanine substitution of two additional conserved residues (K749 and K750) in the region between 735 and 750 (RNase R^K749A/K750A^) resulted in extensive accumulation of non-stop mRNA, suggesting that these residues also played an important role in the selective non-stop mRNA decay activity of RNase R (**Figure 3A**). Collectively, these data implied that conserved residues E740–K741 and K749–K750 play a crucial role in the *trans*-translation mediated decay of non-stop mRNA.

To obtain a more quantitative measure of the non-stop mRNA degradation defect of the alanine substitution mutants, we selected three double-alanine substitution variants (RNase R^T737A/R739A^, RNase R^E740A/K741A^, and RNase R^K749A/K750A^) for further evaluation. First, we examined the relative differences in the steady state abundance of non-stop mRNA among these variants, using RNase R^WT^ and RNase R^723^ as controls (**Figure 3A**). This analysis showed no significant difference in the ability of RNase R^WT^ and RNase R^T737A/R739A^ in the selective degradation of the λ-cI-NS non-stop mRNA. In contrast, alanine substitution variants RNase R^E740A/K741A^ and RNase R^K749A/K750A^ consistently exhibited a 2.5 to 3-fold defect in degradation of the λ-cI-NS reporter; a defect that was statistically significant and similar to the defect exhibited by RNase R^723^ (**Figure 3A**). To eliminate the possibility that the observed non-stop mRNA stability defects of these RNase R variants were due to difference in their relative abundance, we compared their steady state levels to RNase R^WT^ and RNase R^723^ under identical experimental conditions. This analysis revealed no significant differences in the steady state levels of these RNase R variants (**Figure 3B**), suggesting expression or stability difference could account for the observed non-stop mRNA decay defects exhibited by these RNase R variants.

**FIGURE 4:**
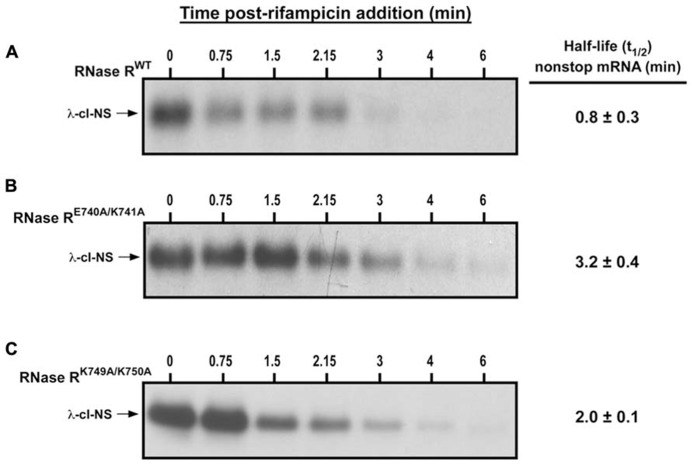
**Non-stop mRNA half-life increase in the presence of RNase R^**E740A/K741A**^ and RNase R^**K749A/K750A**^.** Cells co-expressing λ-cI-NS with the indicated plasmid-borne RNase R variants were harvested at the indicated time points after the addition of rifampicin. Total RNA samples isolated from equal number of cells were subjected to Northern analysis. Intensities of the bands corresponding to the λ-cI-NS reporter were quantified using ImageJ. Non-stop mRNA half-life was calculated by linear regression analysis using the Prism software. The data are representative of three independent experiments (MEAN ± SEM). **(A)** Non-stop mRNA is degraded rapidly (*t*_1/2_ = 0.8 ± 0.3 min) in the presence of RNase R^WT^. **(B)** RNase R^E740A/K741A^ is considerably defective in non-stop mRNA degradation (*t*_1/2_ = 3.2 ± 0.4 min). **(C)** RNase R^K749A/K750A^ exhibited moderate defect in the degradation of non-stop mRNA (*t*_1/2_ = 2.0 ± 0.1 min).

Next, we measured the *in vivo* decay rate and half-life (*t*_1/2_) of the λ-cI-NS non-stop mRNA in the presence of RNase R^WT^, RNase R^E740A/K741A^, and RNase R^K749A/K750A^ (**Figure 4**). Consistent with previous studies, RNase R^WT^ degraded the λ-cI-NS report transcript very efficiently, with a *t*_1/2_ of 0.8 ± 0.3 min (**Figure 4A**). RNase R^E740A/K741A^ had a strong defect in the non-stop mRNA degradation, increasing *t*_1/2_ by roughly 4-fold to 3.2 ± 0.4 min (**Figures 4A,B**). We found this deficiency in non-stop mRNA decay reminiscent of the defect observed with the RNase R^723^ truncation variant. Similarly, in the presence of RNase R^K749A/K750A^, we detected a substantial increase in the stability of the non-stop reporter mRNA with a t_1/2_ of 2.0 ± 0.1 min (**Figure 4C**). Based on these findings, we conclude that residues E740–K741 and K749–K750 in the K-rich domain of RNase R play important but distinct roles in the selective degradation of non-stop mRNA.

### MUTATIONS IN THE K-rich DOMAIN OF RNase R DO NOT AFFECT ITS CATALYTIC ACTIVITY *IN VITRO*

**FIGURE 5:**
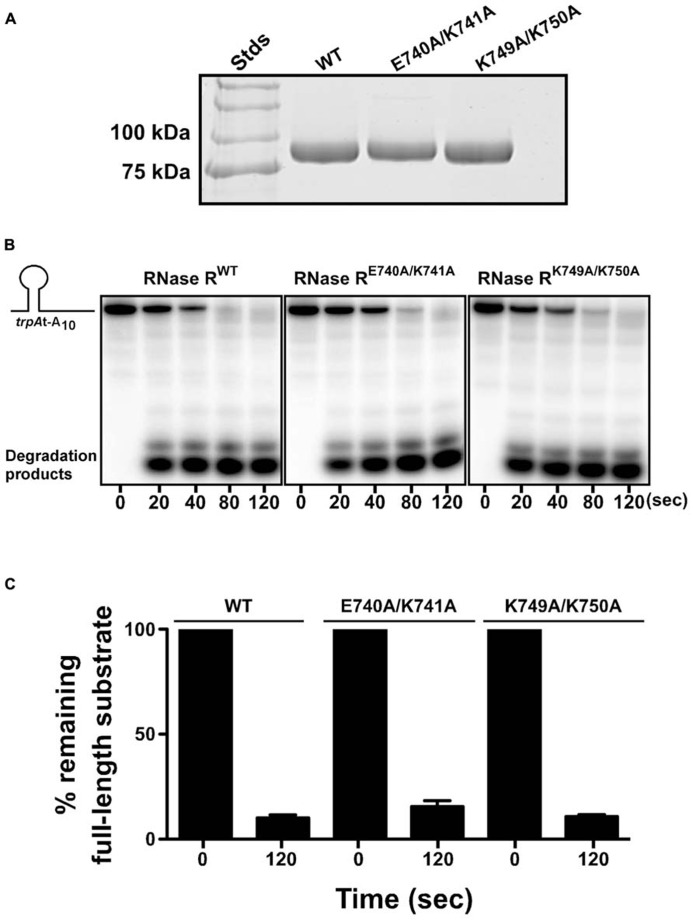
**RNase R variants degrade RNA substrates *in vitro*.** **(A)** Coomassie stained protein gel showing purified RNase R variants. RNase R and its variants (RNase R^E740A/K741A^ and RNase R^K749A/K750A^) were purified from an *rnr*^-^ rnb^-^ strain, resolved by electrophoresis on a 10% SDS-polyacrylamide gel, and stained with Coomassie Brilliant blue.** (B)** Representative autoradiographs showing the degradation of *trp*At-A_10_ RNA substrate by indicated RNase R variants. The ^32^P-labeled RNA substrate was used to test the degradation capability of RNase R^E740A/K741A^ and RNase R^K749A/K750A^. Purified RNase R variants were incubated with the RNA substrate at 37^°^C. The reactions were stopped at indicated times, using denaturing RNA loading dye, and resolved by electrophoresis on denaturing polyacrylamide gel. **(C)** The autoradiographs obtained were used to quantify the amount of full-length RNA substrate present at the start (0 s) and the end (120 s) of the reaction. The data are representative of three independent experiments (MEAN ± SEM).

One possible mechanistic explanation for the observed non-stop mRNA decay defect of RNase R^E740A/K741A^ and RNase R^K749A/K750A^ could be that these residues (K740–K741 and K749–K750) interact directly with the catalytic core of RNase R to modulate its ribonuclease activity. To assess this possibility, we examined the exoribonuclease activity of each alanine substitution variant in an *in vitro* RNA degradation assay. To this end, we overexpressed RNase R^WT^, RNase R^E740A/K741A^, and RNase R^K749A/K750A^, in an *rnr*^-^ rnb^-^ strain, and purified each protein to near homogeneity (**Figure 5A**). The ribonuclease activity of each purified enzyme was assessed by its ability to degrade the *trp*At-A_10_ substrate, a 32-nucleotide long structured RNA containing the *trpA*-terminator stem-loop followed by a stretch of 10 adenines at its 3′ end. Degradation reactions were initiated by the addition of the respective RNase R variant and terminated at the indicated time points by the addition of denaturing sample buffer. The reaction products were resolved by electrophoresis on denaturing polyacrylamide gels (**Figure 5B**), with the activity of each RNase R variant indicated by the disappearance of the full-length substrates and appearance of lower-molecular-weight 2–5 nucleotide products. Using this assay, we observed that RNase R^E740A/K741A^ and RNase R^K749A/K750A^ were as proficient in degradation of this structured RNA substrate as RNase R^WT^ (**Figures 5B,C**). This result indicated that alanine substitutions at amino acid pairs E740–K741 and K749–K750 did not compromise the ability of RNase R to unwind and degrade this RNA substrate, implying that these defined alterations did not impact intramolecular interactions of the K-rich domain with the catalytic core but rather had a specific effect on the *trans*-translation dependent activity of RNase R.

### RESIDUES E740–K741 AND K749–K750 ARE ESSENTIAL FOR RNase R ENRICHMENT ON STALLED RIBOSOMES

**FIGURE 6:**
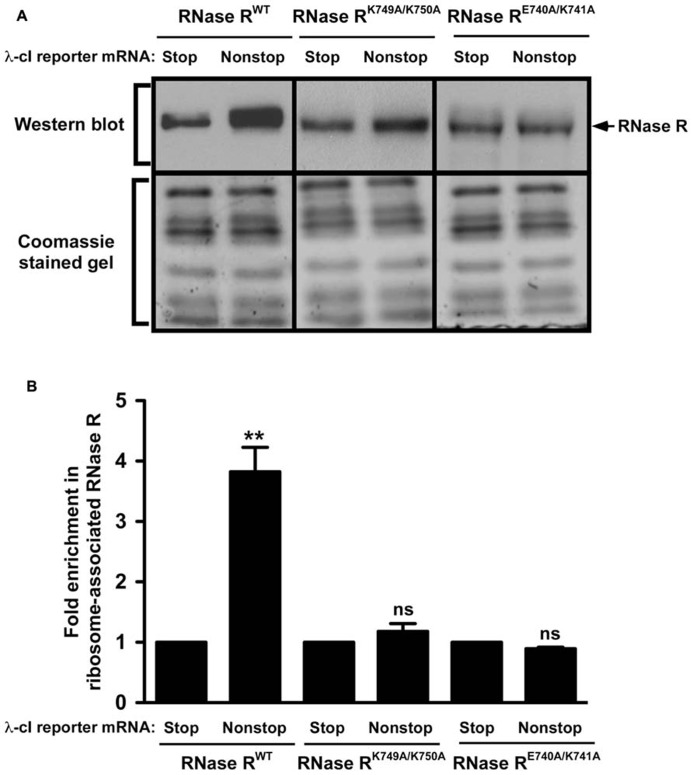
**RNase R variants exhibit defects in enrichment on ribosomes translating non-stop mRNA.** **(A)** Total ribosomes were obtained from cells co-expressing indicated RNase R variants with the λ-cI-S or λ-cI-NS reporter mRNAs. Ribosomes translating the reporter RNAs were isolated from the total ribosomes pool using Ni^2^^+^-NTA affinity chromatography. Equal numbers of ribosomes were resolved by electrophoresis on 10% SDS-polyacrylamide gels. Western blot analysis was performed to detect the presence of RNase R. The intensity of the band corresponding to RNase R was quantified using ImageJ. The top panel shows a representative Western blot, using an RNase R specific antibody, and the bottom panel shows a section of the corresponding Coomassie stained gel displaying equal protein loading.** (B)** The graph represents fold RNase R enrichment on ribosomes translating the λ-cI-NS reporter compared to ribosomes translating the control λ-cI-S reporter. The data are representative of three independent experiments (MEAN ± SEM). *P*-values were calculated by performing student’s *t*-test analysis on the association level of RNase R^WT^ and RNase R^K749A/K750A^ (**P <0.003) or RNase R^WT^ and RNase R^E740A/K741A^ (**P <0.002) on stalled ribosomes translating λ-cI-NS reporter. ns = not statistically significant.

The SmpB-tmRNA system rescues ribosomes stalled at the 3′ end of non-stop mRNAs and recruits RNase R to initiate non-stop mRNA decay (Richards et al., 2006; Ge et al., 2010; Mehta et al., 2012). Our previous studies revealed that the inability of C-terminal truncation variant RNase R^723^ to degrade non-stop mRNA was due to a defect in ribosome enrichment (Ge et al., 2010). This result suggested that the K-rich domain was essential for association of RNase R with rescued ribosomes. We hypothesized that the stabilization of non-stop mRNA in the presence of RNase R^K749A/K750A^ and RNase R^E740A/K741A^ could similarly be due to their inability to productively engage tmRNA-rescued ribosomes. To examine this inference, we used the ribosome enrichment assay (Mehta et al., 2012) to analyze the effect of the K-rich domain alanine substitution variants on the association of RNase R with tmRNA-rescued ribosomes. To ensure the RNase R recruitment process was specific to the λ-cI-NS non-stop transcript, and to verify that we were examining tmRNA-rescued ribosomes, we used the stop codon containing λ-cI-S reporter as negative control, and routinely examined the captured ribosome fractions for the presence and enrichment of SmpB protein. As expected, analysis of the λ-cI-NS reporter in *rnr*^-^ cells expressing the plasmid borne full-length enzyme showed enrichment of RNase R on ribosomes expressing the non-stop reporter (**Figure 6A**). Quantification of ribosome enrichment data, from several independent experiments, showed greater than 3-fold RNase R^WT^ enrichment on ribosome translating the λ-cI-NS non-stop reporter as compared to the λ-cI-S control transcript (**Figure 6B**). In contrast, ribosome enrichment analysis of the RNase R^E740A/K741A^ variant in *rnr*^-^ cells revealed only background level of ribosome association in cells expressing either the λ-cI-NS or λ-cI-S reporter transcript (**Figure 6**). Although we consistently detected a small but reproducible increase in enrichment of the RNase R^K749A/K750A^ variant on stalled ribosomes (**igures 6A,B**), this level of enrichment did not fully reflect the observed increase in non-stop mRNA half-life. We attribute this difference to the fact that the ribosome enrichment assay, which involves several *in vitro* processing steps, is not sensitive enough to detect intermediate level enrichment of RNase R variants, whereas the rifampicin-chase assay is more sensitive in detecting subtle differences in non-stop mRNA degradation rates. Based on these findings, we conclude that the non-stop mRNA decay defect of RNase R^E740A/K741A^ and RNase R^K749A/K750A^ is due to the failure of these variants to productively engage tmRNA-rescued ribosomes.

**FIGURE 7:**
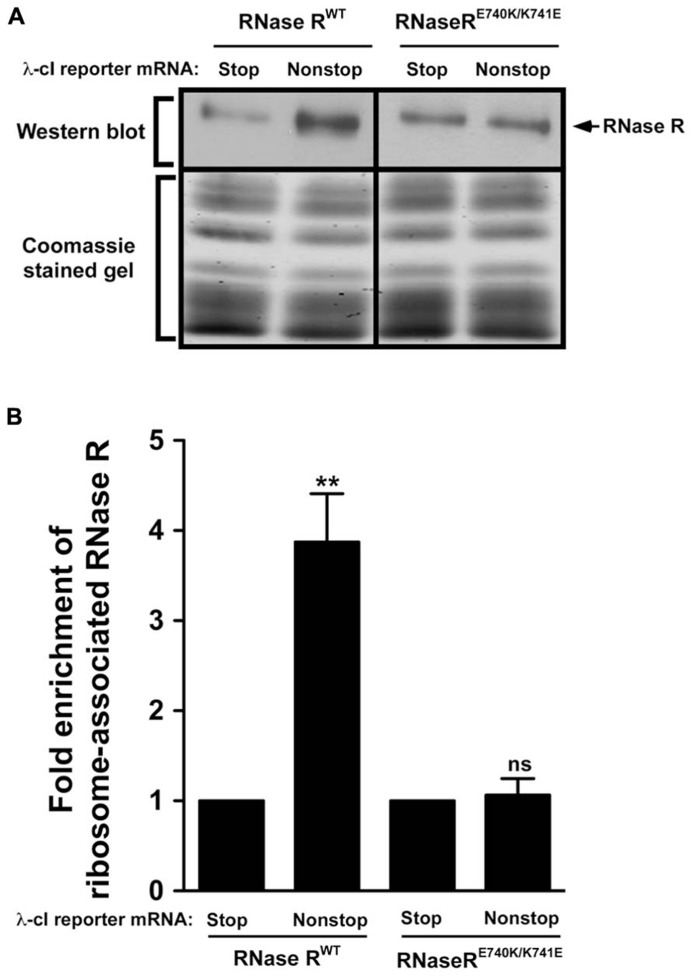
**Swapping of residues E740 and K741 renders RNase R defective in enrichment on stalled ribosomes.** **(A)** Enriched ribosomes were obtained from cells co-expressing RNase R^WT^ or the RNase R^E740K/K741E^ switch variant with either the λ-cI-S or λ-cI-NS reporter mRNA. Western blot analyses were performed as described in **Figure 6**. Intensities of the bands were quantified using ImageJ. The top panel shows a representative Western blot, using an RNase R specific antibody, and the bottom panel shows a section of the corresponding Coomassie stained gel displaying equal protein loading.** (B)** The graph represents fold enrichment of RNase R on stalled ribosomes compared to its corresponding background level with the control λ-cI-S reporter. The data are representative of three independent experiments (MEAN ± SEM). *P*-values were calculated by performing student’s *t*-test analysis on the association level of RNase R^WT^ and RNase R^E740K/K741E^ (**P <0.008) on stalled ribosomes translating λ-cI-NS reporter. ns = not statistically significant.

Alanine substitution at residues E740 and K741 appears to have a profound effect on the *trans*-translation related activity of RNase R. Given the nature of these amino acids, it was conceivable that they interacted with each other, perhaps by forming a salt bridge required for maintaining intramolecular contacts. The presence of these contacts might be important for the non-stop mRNA degradation activity of RNase R. We reasoned that swapping the positions of these residues could preserve these interactions, and hence the *trans-*translation activity of RNase R on stalled ribosomes. To assess this possibility, we constructed the RNase R^E740K/K741E^ switch variant and performed ribosome enrichment assays. This analysis showed that the E–K switch variant (RNase R^E740K/K741E^) had a strong defect in association with tmRNA-rescued ribosomes (**Figure 7**). This finding indicated that the position and orientation of residues E740 and K741 are important for productive engagement of RNase R with the *trans-*translation machinery.

## DISCUSSION

RNase R is a versatile 3′–5′ exoribonuclease with the distinctive ability to unwind and degrade structured RNA substrates without ATP consumption (Cheng and Deutscher, 2002; Vincent and Deutscher, 2009). RNase R does not exhibit sequence specificity and is thus involved in the processing and turnover of a diverse array of cellular RNAs, including mRNA, rRNA, and tRNAs (Cheng and Deutscher, 2003; Jacob et al., 2013). Given the broad range of RNase R functions, it is of high interest to understand its overall architecture and the spatial arrangement of its distinct N- and C-terminal domains. Similarly, given its lack of substrate specificity it is of particular interest to understand how this general exoribonuclease is recruited to stalled ribosomes to carry out selective degradation of non-stop mRNAs. Our explorations into the global architecture of the *E*.* coli *RNase**R have yielded the first glimpses of its overall shape and domain arrangement, indicating a tri-lobed structure with a core catalytic domain that is highly similar to the known crystal structure of RNase II. Our data show that the unique N- and C-terminal domains of RNase R flank the catalytic core of the protein. Consistent with bioinformatics analysis (Matos et al., 2011), our SAXS and homology modeling data suggest that in the absence of any bound partners or substrates the K-rich domain has significant structural flexibility. Our findings are thus consistent with the notion that the K-rich domain is disordered in solution but might adopt a more ordered structure upon binding to interacting partners, such as RNA or ribosomal components.

Recent studies suggest that the K-rich domain confers a variety of properties on RNase R, including enhancing its affinity for RNA, modulating its ability to degrade structured substrates (Matos et al., 2011), and regulating its *in vivo* stability. Our data suggest potential contacts between the catalytic core and the K-rich domain of RNase R. This indication is consistent with the notion that the K-rich region interacts with the core of the enzyme and plays a regulatory role. Characterization of one such contact suggested that acetylation of Lys544 plays a key regulatory role in interactions of the K-rich domain and the catalytic core of the enzyme, with implications for both function and stability of the protein (Liang and Deutscher, 2012). It was proposed that the un-acetylated form of K544 prevents binding of the SmpB-tmRNA complex and as a consequence prevents degradation of RNase R by cellular proteases (Liang and Deutscher, 2012). Although the key components of the *trans*-translation system play a role in destabilization of RNase R, it has not been determined whether this is a direct consequence of the *trans*-translation process. Furthermore, the precise amino acid residues necessary for this intramolecular interaction have not been identified.

Previous work from our lab established that the unique K-rich domain of RNase R is required for the *trans-*translation mediated decay of non-stop mRNAs. These studies also suggested that some of the key functional amino acids reside in the region between residues 735 and 750 of the K-rich domain. Here, we presented a detailed analysis of the contributions of this functionally important region of the K-rich domain to the *trans*-translation process. We have identified key amino acid residues (E740, K741, K749, and K750) in the K-rich domain that are involved in the selective and SmpB-tmRNA dependent elimination of non-stop mRNA. Alanine substitution of residues E740 and K741 significantly weaken the recruitment of RNase R to tmRNA-rescued ribosomes and as a consequence diminish its ability to selectively degrade the associated non-stop mRNA. In addition, simultaneous mutation of residues K749 and K750 renders RNase R markedly defective in non-stop mRNA decay. It is interesting to note that the effect of these alanine substitutions is specific to the *trans*-translation related activity of RNase R, as they do not compromise its catalytic activity. These four conserved residues, particularly E740 and K741, might exert their influence on the activity of RNase R by participating in decisive intra- or intermolecular interactions. Our data suggest that these amino acids most likely participate in intermolecular interactions with components of the rescued ribosome. We proposed that ribosomal RNA or ribosomal proteins might interact directly with these residues, thereby enabling RNase R to productively engage stalled ribosomes. Taken together, these investigations have shed new light on the global architecture and the relative spatial arrangement of the N- and C-terminal domains of RNase R and highlight the functional significance of key conserved residues in the *trans-*translation related activities of this versatile enzyme.

## Conflict of Interest Statement

The authors declare that the research was conducted in the absence of any commercial or financial relationships that could be construed as a potential conflict of interest.
